# Genetic evaluation of pediatric pituitary adenomas and *USP8*-related genotype-phenotype correlations in Cushing’s disease

**DOI:** 10.1007/s11102-025-01557-6

**Published:** 2025-08-14

**Authors:** Rida Zainab, Sukhvir Kaur, Justin Lack, Morgan Similuk, Mayank Tandon, Rajarshi Ghosh, Bryce A. Seifert, Mari Tokita, Chelsi Flippo, Jia Yan, Magdalena Walkiewicz, Prashant Chittiboina, Christina Tatsi

**Affiliations:** 1 Unit on Hypothalamic and Pituitary Disorders, Eunice Kennedy Shriver National Institute of Child Health, and Human Development (NICHD), National Institutes of Health, Bethesda, MD USA; 2 Collaborative Bioinformatics Resource, National Institute of Allergy and Infectious Diseases (NIAID), National Institutes of Health, Bethesda, United States; 3 Centralized Sequencing Program, National Institute of Allergy and Infectious Diseases (NIAID), National Institutes of Health, Bethesda, MD USA; 4 Division of Pediatric Endocrinology, Children’s National Hospital, Washington, DC USA; 5 Neurosurgery Unit for Pituitary and Inheritable Diseases, National Institute of Neurological Disorders and Stroke (NINDS), National Institutes of Health, Bethesda, MD USA

**Keywords:** Pediatric pituitary adenomas, Corticotroph tumor, Somatotroph tumor, Cushing, Gigantism

## Abstract

**Purpose:**

Pituitary adenomas (PAs) constitute a rare pediatric diagnosis and their pathogenetic mechanisms are not clearly understood. The aim of this study was to evaluate the prevalence of genetic defects in pediatric PAs through germline and tumor testing, and to describe genotype-phenotype correlations.

**Methods:**

Fifty-four pediatric patients with PAs and available germline and/or tumor samples were studied. Germline and/or tumor sequencing were reviewed for variants in genes previously associated with pituitary tumorigenesis.

**Results:**

Germline genetic testing revealed a pathogenic variant in *AIP* gene in 2 patients with growth hormone excess (GHE) and a likely pathogenic variant in *CDKN2A* in a patient with Cushing’s disease (CD). Somatic gene sequencing identified pathogenic variants in *GNAS* in 4/7 patients (57.1%) with GHE. 6/38 patients (15.8%) with CD had pathogenic variants in *USP8* gene, and in one tumor pathogenic variants in *PRKAR1A*, *TP53* and *MEN1* genes were identified. Overall, pathogenic/likely pathogenic germline or somatic variants were identified in 14/54 patients (25.9%). When evaluating the genotype-phenotype correlations in patients with CD, patients with somatic *USP8* pathogenic variants had larger tumors (median size: 9.5 mm [6.5, 13.3] vs. 6 mm [4.0, 7.0], *p* = 0.048), trend towards higher incidence of cavernous sinus invasion (50% vs. 12.5%, *p* = 0.06), and higher risk of non-remission after surgery (33.3% vs. 0%, *p* = 0.021) compared to patients without *USP8* variants.

**Conclusions:**

Somatic *USP8* pathogenic variants correlate with worse tumor behavior and patient outcomes in pediatric-onset CD. Unlike GH-secreting PAs, the genetic basis of the majority of pediatric corticotroph PAs remains unclear. Further studies are needed to explore the genetic drivers of pediatric CD.

**ClinicalTrials.gov ID:**

NCT00001595, NCT03206099.

**Supplementary Information:**

The online version contains supplementary material available at 10.1007/s11102-025-01557-6.

## Introduction

Pediatric pituitary adenomas (PAs) especially hormone-secreting, such as corticotroph PAs leading to adrenocorticotropin hormone (ACTH) excess causing Cushing’s disease (CD) or somatotroph PAs leading to growth hormone (GH) excess (GHE) causing gigantism/acromegaly, are rare pediatric endocrine conditions [1–3].

The description of genetic causes of PAs may provide information on pathways of pituitary tumorigenesis and therapeutic targets. Germline genetic defects however explain only a small proportion of adult cases [4]. These are often gene defects in the context of multiple endocrine neoplasia syndrome type 1 (MEN1 caused by *MEN1* gene defects), MEN4 (caused by *CDKN1B* gene defects), *AIP* gene defects leading to familial isolated pituitary adenoma (FIPA) syndrome, Carney complex (caused by *PRKAR1A* gene defects) and other rare causes [5]. Somatic gene defects often provide more information about the pathogenesis of these tumors and may explain up to 50–60% of adult cases depending on the type of tumor and the population studied [4]. Specifically, *USP8* and, less commonly, *USP48*, *TP53*, and *BRAF* somatic gene defects have been associated with CD, while *GNAS* somatic gene defects with GH-secreting PAs [6]. In the pediatric population specifically, few studies have been published on the genetic findings of these patients suggesting that a low prevalence of germline and somatic gene defects occur in pediatric CD, while higher frequency is noted in pediatric GHE [6–8].

The identification of genetic causes of PAs may provide guidance on screening for other related manifestations of the associated genetic syndrome, such as for cardiac myxomas in patients with Carney complex. Additionally, some genetic findings may predispose to a more or less aggressive phenotype and predict their response to treatment [9]. For example, a previous study suggested that children with *USP8* somatic mutations have higher risk of non-remission or recurrence, while tumors with large copy number variations (CNVs) are larger and predict lower remission rates [6, 10]. Thus, exploring the genetic background of these tumors may also provide molecular markers of disease prognosis.

In this study, we describe the genetic profile of pediatric corticotroph and somatotroph tumors in a large cohort of pediatric patients with extensive germline and somatic DNA sequencing, and expand the genotype-phenotype correlations. The analysis of novel candidate genes is underway and not the focus of this study.

## Methods

### Subjects

Fifty-four pediatric patients with confirmed PAs and available germline and/or tumor DNA samples were enrolled in the study. Patients were recruited between 2021 and 2024 at the Clinical Center (CC) of the National Institutes of Health under the protocol 97-CH-0076 (NCT00001595) and 17-I-0122 (NCT03206099). The diagnosis of corticotroph (*n* = 47) and somatotroph (*n* = 7) tumors was based on previously defined criteria and confirmed with histologic evaluation in most cases [11, 12]. Most patients were evaluated at the time of initial diagnosis, but some were enrolled at the time of recurrent disease or at regular follow-up visits. Remission was defined as postoperative cortisol < 2mcg/dL in patients with CD or normalization of IGF-1 within 3 months after surgery for patients with GHE. Informed consent was obtained from parents and assent from patients if developmentally appropriate. All study procedures were approved by the *Eunice Kennedy Shriver* National Institute of Child Health & Human Development (NICHD) and the National Institute of Allergy and Infectious Disease (NIAID) Institutional Review Board (IRB).

### Germline genome sequencing

Germline DNA was extracted from peripheral blood leukocytes. Whole genome sequencing (WGS) data was generated for samples at the Baylor College of Medicine Human Genome Sequencing Center Clinical Laboratory (HGSC-CL) using established library preparation and sequencing methods. Libraries were prepared using KAPA Hyper PCR-free library reagents (KK8505, KAPA Biosystems Inc.) on Beckman robotic workstations (Biomek FX and FXp models). Briefly, DNA (750 ng) was sheared into fragments of approximately 200–600 bp using the Covaris E220 system (96 well format, Covaris, Inc. Woburn, MA) followed by purification of the fragmented DNA using AMPure XP beads. A double size selection step was employed, with different ratios of AMPure XP beads, to select a narrow size band of sheared DNA molecules for library preparation. DNA end-repair and 3’-adenylation were then performed in the same reaction followed by ligation of the Illumina unique dual barcodes adapters (Cat# 20022370) to create PCR-Free libraries, and the library was run on the Fragment Analyzer (Advanced Analytical Technologies, Inc., Ames, Iowa) to assess library size and presence of remaining adapter dimers. This was followed by qPCR assay using KAPA Library Quantification Kit (KK4835) using their SYBR^®^ FAST qPCR Master Mix to estimate the size and quantification. WGS libraries were sequenced on the Illumina NovaSeq 6000 instrument on S4 flow cells to generate 150 bp paired-end reads. Confirmation of potentially relevant findings was performed using capillary sequencing or other appropriate method. The read depth of germline WGS was set at 30x. For the list of genes of interest average depth was >30x except for one sample where depth was 20-30x and two genes (*USP8* and *VHL*) which had depth 10–43x.

Four patients had clinical germline whole exome sequencing at an outside facility (GeneDx, Germantown, MD) with negative results. In these four patients, somatic tumor sequencing identified a hotspot variant and thus germline testing was not repeated and is herein reported as negative but review of raw germline data was not available. In five additional patients, only tumor WGS analysis was performed because germline sample was not available or has been depleted. In four of these tumors, no variant in the list of genes of interest was identified and thus germline analysis was presumed negative. In one patient where only tumor WGS analysis was performed, two pathogenic variants were identified. Since germline sample was not available we could not confirm whether any of these variants were also present in the germline and the patient is included only in the somatic gene results.

### Tumor sequencing

Tumor DNA was extracted from fresh frozen tissue samples using the Quick-DNA Microprep Plus Kit (Zymo Research, Cat.no.4074) or from formalin-fixed, paraffin embedded tissue using the Pinpoint Slide DNA isolation system kit (Zymo Research, Cat.no.D3001). Wherever possible tumor tissue was selected either with macroscopic inspection of the fresh frozen samples or with microscopic selection of the tumor area on tissue slides. However, contamination with normal surrounding tissue at low concentration cannot be excluded. Samples were screened with Sanger sequencing for the presence of hotspot pathogenic variants in *USP8* gene (codons 713–720) for corticotropinomas, or *GNAS* gene (codons 201 and 227) for somatotropinomas. Thirty-one samples (corticotropinomas = 27, somatotropinomas = 4) were further analyzed with WGS. Uniquely indexed paired-end libraries of genomic DNA (gDNA) were prepared using Illumina^®^ TruSeq^®^ Nano DNA Library Prep kits. 100 ng of genomic DNA was fragmented to a 400 bp insert size on the Covaris which generated dsDNA fragments with 3’ or 5’ overhangs. The sheared DNA was blunt-ended and library size selection was performed using sample purification beads. A single ‘A’ nucleotide was added to the 3’ ends of the blunt fragments to prevent them from ligating to each other during the adapter ligation reaction. A corresponding single ‘T’ nucleotide on the 3’ end of the adapter provided a complementary overhang for ligating the adapter to the fragment. The indexed adapters were ligated to the ends of the DNA fragments and then PCR-amplified to enrich for fragments that have adapters on both ends. The final purified product was quantitated by qPCR before cluster generation and set up on S2 flowcell on NovaSeq 6000 for a paired end 150 cycles sequencing. The read depth of tumor WGS was set at 60x. For the list of genes of interest average depth was > 60x in 79% of gene-samples, and > 30x in 99.5% gene-samples. One sample had low coverage of *TBX19* gene.

### Bioinformatic analysis

Raw fastq files were trimmed for quality and adapter contamination using Trimmomatic v0.39 and mapped to the GRCh38 human reference genome using bwa-mem [13, 14]. Samblaster v0.1.26 (Faust and Hall 2014) was used to mark PCR duplicates and resulting BAM files were then used to call variants following the GATK Best Practices using GATK4 v4.3.0.0 [15, 16]. 

For this project, we reviewed variants in a panel of genes (*n* = 66) with possible association with pituitary tumorigenesis (either as drivers of PAs or contributing to the severity of their presentation based on literature search, Supplementary Table 1). Germline genome analysis was performed using a custom-enhanced analysis tool (SEQR) [17]. Variants were filtered if they were rare in the public databases (https://gnomad.broadinstitute.org/, last accessed: 05/05/2024) with allele frequency < 0.01 (< 1%) and had possible structural or splice site effect on the translated protein. Variants were confirmed with direct visualization in IGV browser. In silico prediction of the effect of variants on the structure and function of the corresponding proteins was performed with various bioinformatic tools as appropriate [MutationTaster (http://www.mutationtaster.org/), Polymorphism Phenotyping v2 (PolyPhen-2) (http://genetics.bwh.harvard.edu/pph2), SIFT (Sorting Tolerant From Intolerant) (http://sift.jcvi.org), FATHMM (Functional Analysis through Hidden Markov Models v2.3) (http://fathmm.biocompute.org.uk/), SpliceAI (https://spliceailookup.broadinstitute.org/) and SpliceRover (http://bioit2.irc.ugent.be/rover/)]. Germline variants were classified based on the American College of Medical Genetics and Genomics (ACMG) guidelines [18, 19] and the nomenclature of the identified variants is consistent with the Human Genome Variation Society (HGVS) guidelines. Variants that were considered pathogenic, likely pathogenic, or variants of uncertain significance (VUS) are reported in the results.

### Statistical analysis

Categorical data are presented as counts (proportions) and were compared between groups using Fisher’s exact test. Continuous data were checked for normality and continuous data with normal distribution are described as mean (standard deviation) and were compared between groups using student’s t-test. Continuous data without normal distribution are presented as median [first quartile, third quartile] and were compared between groups using Wilcoxon rank-sum test. Probability of recurrence free survival and hazard ratio (HR) was performed with cox proportional hazard regression and presented as HR and 95% confidence intervals (CI). Results were considered significant if *p*-value was < 0.05. Statistical analyses were performed in R.

## Results

### Cohort characteristics

Overall, 54 patients were included in the study (Table 1). The mean age at diagnosis was 12.9 years (3.2) and 27 patients (50%) were female. Patients were either diagnosed with CD (*n* = 47) or GHE (*n* = 7). Most of the patients with CD had microadenomas (median tumor size: 6.0 mm [4.0, 7.8]), while all patients with GHE had macroadenomas (median tumor size: 22.0 mm [16, 31]). After surgery, remission was documented in 45 of 47 patients with CD (95.7%), and in 3 of 7 patients with GHE (42.9%).

**Table 1:** Cohort characteristics

	CD (*N* = 47)	GHE (*N* = 7)	Overall (*N* = 54)
**Sex**			
Female	23 (48.9%)	4 (57.1%)	27 (50.0%)
Male	24 (51.1%)	3 (42.9%)	27 (50.0%)
**Race**			
Asian	2 (4.3%)	2 (28.6%)	4 (7.4%)
Black/African American	2 (4.3%)	0 (0%)	2 (3.7%)
Multiracial	4 (8.5%)	1 (14.3%)	5 (9.3%)
White	35 (74.5%)	4 (57.1%)	39 (72.2%)
Unknown	4 (8.5%)		4 (7.4%)
**Ethnicity**			
Hispanic or Latino	10 (21.3%)	1 (14.3%)	11 (20.4%)
Not Hispanic or Latino	33 (70.2%)	6 (85.7%)	39 (72.2%)
Unknown	4 (8.5%)		4 (7.4%)
**Age at diagnosis (years)**	13.0 (3.1)	12.0 (4.5)	12.9 (3.2)
**Disease duration (years)**	2.5 [1.5, 3.9]	2.8 [2.1, 3.0]	2.5 [1.5, 3.8]
Midnight serum cortisol (mcg/dL)*	13.4 [10.5, 17.3]	NA	13.4 [10.5, 17.3]
UFC (fold change from ULN)*	4.04 [2.58, 6.76]	NA	4.04 [2.58, 6.76]
**IGF1 z-score** ^**#**^	NA	4.79 [3.46, 8.83]	4.79 [3.46, 8.83]
**Tumor size (mm)**	6.0 [4.0, 7.8]	22.0 [16.0, 31.0]	6.0 [4.0, 9.0]
**Cavernous sinus invasion**	7 (14.9%)	4 (57.1%)	11 (20.4%)
**Remission after surgery**	45 (95.7%)	3 (42.9%)	48 (88.9%)
**Recurrence after initial remission**	6 (13.3%)	1 (33.3%)	7 (14.6%)

^*^For patients with Cushing’s disease, ^#^For patients with growth-hormone excess

CD: Cushing’s disease; GHE: Growth-hormone excess; UFC: Urinary free cortisol; ULN: Upper limit of normal; IGF-1: Insulin-like growth factor 1

### Germline genetic testing

**Fig. 1:**
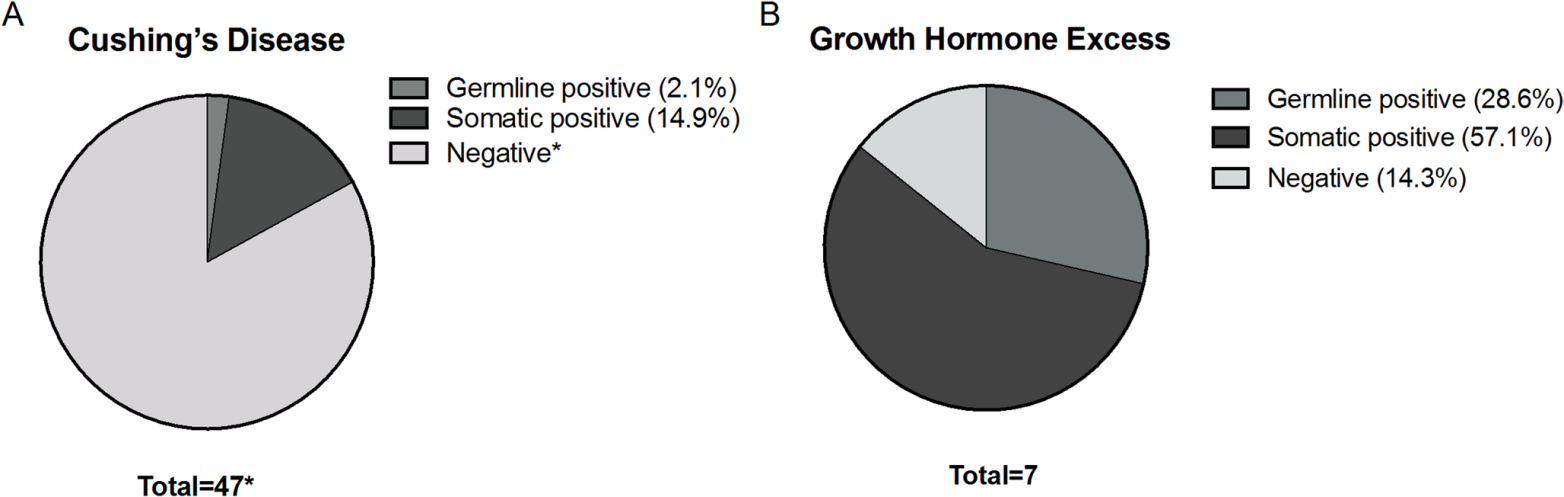
Yield of pathogenic/likely pathogenic germline and somatic variants in pediatric patients with Cushing’s disease (**A**) and growth hormone excess (**B**). *Some patients did not have both germline and tumor analysis available

Genetic testing revealed a germline pathogenic/likely pathogenic variant in 3/53 samples (5.7%). Two patients with GHE had pathogenic variants in *AIP* gene, and one patient with CD had a likely pathogenic variant in *CDKN2A* gene (Table 2; Fig. 1).

**Table 2:** List of variants (pathogenic, likely pathogenic or variants of uncertain significance) identified in germline and tumor genetic testing

Case	Diagnosis	Sex	Germline variant			Tumor variant	
			Gene	Variant	ACMG Variant Classification	Gene	Somatic variant
1	CD	Male	*MSH6*	c.3259C>G, p.Pro1087Ala	VUS	None	
2	CD	Female	*CABLES1*	c.985C>T, p.Arg329Trp	VUS	None	
3	CD	Female	*SDHC*	c.98C>T, p.Thr33Met	VUS	None	
			*TSC2*	c.1816A>G, p.Ile606Val	VUS	None	
4	CD	Male	*MSH2*	c.557A>G, p.Asn186Ser	VUS	None	
5	CD	Female	*RB1*	c.929G>A, p.Gly310Glu	VUS	None	
6	CD	Female	*AIP*	c.145G>A, p.Val49Met	VUS	*USP8*	c.2152T>C, p.Ser718Pro
7	CD	Female	None			*USP8*	c.2152T>C, p.Ser718Pro
8	CD	Male	None			*USP8*	c.2155_2157del, p.Ser719del
9	CD	Male	*MAX*	c.25G>T, p.Val9Leu	VUS	None	None
10	CD	Female	None			*USP8*	c.2155_2157del, p.Ser719del
11	CD	Female	*CABLES1*	c.1555C>G, p.Leu519Val	VUS	None	
12	CD	Female	*CDKN2A*	c.146T>C, p.Ile49Thr	Likely pathogenic	None	
			*DICER1*	c.5762A>G, p.Asn1921Ser	VUS	None	
13	CD	Female	None			*USP8*	c.2155_2157del, p.Ser719del
14	CD	Male	*RB1*	c.367A>G, p.Asn123Asp	VUS	Not available	
15	CD	Male	Not available			*PRKAR1A*	c.55G>T, p.Glu19Ter
						*TP53* *MEN1*	c.536A>G, p.His179Arg c.1357C>T, p.Gln453Ter
16	CD	Male	*AIP*	c.220G>A, p.Glu74Lys	VUS	None	
17	GHE	Male	None			*GNAS*	c.601C>T, p.Arg201Cys
18	GHE	Male	None			*GNAS*	c.680A>T, p.Gln227Leu
19	GHE	Male	*AIP*	c.811C>T, p.Arg271Trp	Pathogenic	None	
20	GHE	Female	None			*GNAS*	c.601C>T, p.Arg201Cys
21	GHE	Female	*AIP*	c.241C>T, p.Arg81Ter	Pathogenic	None	
22	GHE	Female	None			*GNAS*	c.602G>A, p.Arg201His

ACMG: American College of Medical Genetics and Genomics; CD: Cushing’s disease; GHE: Growth hormone excess; VUS: Variant of uncertain significance

The variants in *AIP* gene were identified in patients with GHE who presented with macroadenomas. The younger patient had a more aggressive tumor that required multiple surgeries, radiation and medical treatment, while the second patient is currently in remission after two surgical resections. Tumor analysis in both patients revealed loss of the wild-type allele at the tumor level confirming loss of heterozygosity (LOH).

The variant in *CDKN2A* gene (c.146T>C, p.Ile49Thr) was identified in a 15-year-old boy with CD who presented with a microadenoma (4 mm) and typical features of hypercortisolemia. He underwent successful transsphenoidal surgery and remains normocortisolemic one year after surgery. Tumor analysis with ddPCR for somatic CNV did not reveal LOH of the *CDKN2A* locus at the tumor level. However, due to limited tumor DNA availability, no further molecular testing was possible on the tumor sample.

Variants of uncertain significance (VUS) were noted in genes associated with PAs in additional patients (Table 2). The effect of these variants has not yet been reported in functional studies and further classification was not possible at this time.

### Tumor genetic testing

Tumor samples were available for hotspot gene mutation screening in most patients (*n* = 45; CD = 38, GHE = 7). In 31 samples, tumor WGS was also performed. In the remaining 9 patients for whom germline genetic test was performed, tumor samples were not available for somatic genetic testing. Overall tumor pathogenic variants were detected in 11/45 samples (24.4%).

We identified somatic pathogenic variants in *GNAS* gene in 4/7 patients with GHE (57.1%) and somatic pathogenic variants in *USP8* gene in 6/38 patients with CD (15.8%) (Table 2; Fig. 1). Of the 28 tumor samples with WGS analysis, review for somatic pathogenic variants in the select list of genes identified one additional sample with three pathogenic variants: one in each of *MEN1*, *TP53* and *PRKAR1A* gene; no germline sample was available for this patient. No other pathogenic variants in *TP53*, *USP48* and *BRAF* were identified.

### Genotype-phenotype correlations in patients with *USP8*-positive Cushing’s disease

**Fig. 2:**
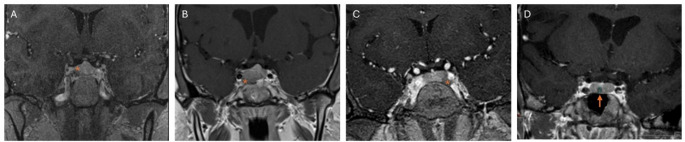
(**A**-**C**) Representative post-contrast magnetic resonance imaging (MRI) studies of pituitary corticotropinomas in three patients with Cushing’s disease and *USP8* pathogenic variants showing larger tumors with signs of cavernous sinus invasion (asterisk). (**D**) Representative post-contrast MRI study of a patient with Cushing’s disease without *USP8* pathogenic variant showing a typical microadenoma (arrow)

**Fig. 3:**
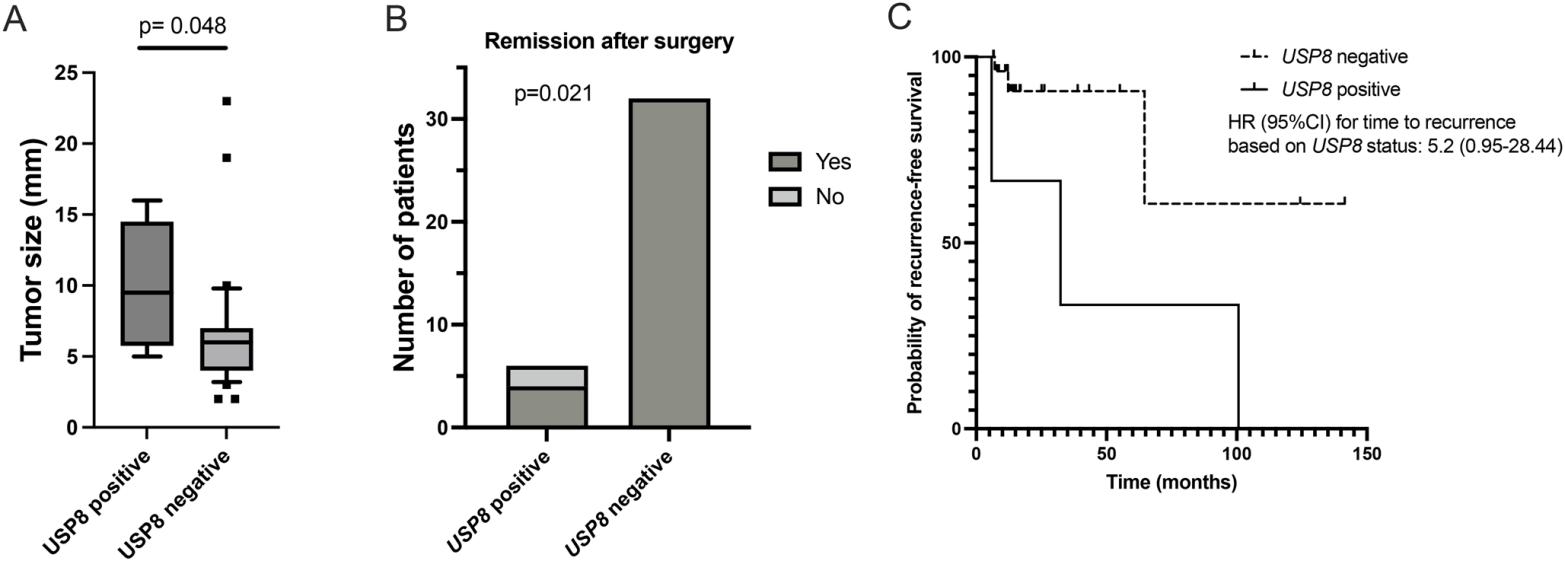
(**A**) Tumor size in patients with Cushing’s disease with or without *USP8* pathogenic variants (line shows median and whiskers 10-90th percentile). (**B**) Remission after surgery in patients with Cushing’s disease with or without *USP8* mutation. (**C**) Time-to-event plot of probability of recurrence based on *USP8* status

Since *USP8* somatic variants were the most commonly identified gene defect in pediatric CD tumors, we investigated the phenotype correlations in pediatric CD cohort based on the presence or absence of a *USP8* variant. Patients with CD and *USP8* somatic pathogenic variants had similar age at diagnosis, sex distribution, disease duration, and biochemical markers of hypercortisolemia (midnight serum cortisol, 24 h urinary free cortisol, and plasma ACTH) compared to patients without *USP8* pathogenic variants (*p* > 0.05). However, patients with *USP8* pathogenic variants had larger tumors (median tumor size: 9.5 mm [6.5, 13.3] vs. 6.0 mm [4.0, 7.0] in patients without *USP8*, *p*= 0.048), and they had higher chance of having macroadenomas (50.0%) compared to those without *USP8* variants (9.4%, *p*= 0.039) (Fig. 2). Patients with *USP8* pathogenic variants also tended to have higher incidence of cavernous sinus (CS) invasion (50.0% vs. 12.5% in patients without *USP8*, *p*= 0.06), and had higher risk of non-remission after surgery (33.3% vs. 0% in patients without *USP8*, *p*= 0.021) (Fig. 3). Finally, patients with *USP8* positive tumors and initial remission had higher incidence of recurrence (75.0%) vs. *USP8* negative tumors (9.4%, *p*= 0.01), which was also suggested in a time-to-event analysis though further follow-up is needed to draw definite conclusions (HR for *USP8*-positive: 5.2, 95%CI: 0.95-28.44, Fig. 3). Thus, patients with *USP8* positive tumors had overall worse prognosis most commonly due to larger tumors that tended to invade the cavernous sinus and recur even after initial remission.

## Discussion

The description of the genetic causes of PAs will enhance our understanding on the mechanisms of pituitary tumorigenesis and provide insight on the disease presentation. However, we here describe that our knowledge of the genetic background of corticotroph PAs, remains limited and known germline and somatic gene defects explain only one fourth of pediatric cases. However, when identified, specific genetic defects correlate with disease presentation and provide prognostic information on patient outcomes. In our analysis, germline genetic testing had low yield on identifying genetic causes of PAs, especially in pediatric onset CD. Amongst the pathogenic/likely pathogenic variants, the *AIP* gene variants (c.811 C > T, p.Arg271Trp; and c.241 C > T, p.Arg81Ter) have been previously reported in patients with GHE, and functional studies have confirmed their pathogenic effects on the translated protein [20–22]. On the contrary, *CDKN2A* gene variants have not been previously found in patients with CD. The variant we identified (c.146T > C, p.Ile49Thr) has been reported in pancreatic cancer and melanoma, and functional studies reported an effect of cell-cycle regulation [23]. Previous reports of inactivation of *CDKN2A* in PAs along with the high expression of *CDKN2A* in normal pituitary gland (based on GTEx data) support a role of the *CDKN2A* variant in pituitary tumorigenesis [24]. Other cyclin-dependent kinases, such as *CDKN1B*, have been reported in PAs, and cell-cycle dysregulation is one of the main mechanisms of pituitary and other tumors [25, 26]. Although a second “hit” at the tumor level could not be confirmed, this could be due to the limited tissue availability and further analyses or additional cases may help in this direction. The remaining germline variants were classified as VUS suggesting that further studies are needed to characterize their functional effect. Some of these were found in patients for whom an alternative genetic driver of tumorigenesis was reported. For example, a patient with CD carried a germline VUS in *AIP* gene; although the variant was previously reported in a cohort of patients with somatotropinomas, functional studies have shown conflicting evidence regarding its effect on protein function, *AIP* pathogenic variants in corticotropinomas are rare, and the patient also carried a somatic *USP8* pathogenic variant [20, 27, 28]. VUS were also noted in genes involved in the DNA mismatch repair system (*MSH2*, *MSH6*), in tuberous sclerosis syndrome (*TSC2*), the 3P association (3PAs) (*SDHC*, *MAX*) and other genes associated with PAs in rare cases [29–31]. 

In both CD and GHE, somatic events were more common. In previous studies in adults, *USP8* somatic pathogenic variants were present in 40–60% of patients with CD, while in a previous pediatric study, *USP8* pathogenic variants were found in 31% of patients [6, 32, 33]. Herein, we found *USP8* pathogenic variants in 15.8% of our samples supporting that *USP8* gene defects do not explain the majority of pediatric corticotropinomas. However, we were able to expand the genotype-phenotype correlations of *USP8* variants in pediatric CD. In a previous pediatric study, *USP8* variants were associated with higher risk of non-remission or recurrence (grouped together), but not with other disease findings [6]. On the contrary, in adults *USP8* variants are associated with smaller tumors, and studies on long term remission suggest conflicting data [33–35]. We here report that pediatric *USP8* positive tumors are larger than wild-type and have higher risk of CS invasion leading to higher risk of non-remission after surgery. The long-term prognosis of these patients remains to be seen, and we acknowledge the potential limitations of the small sample size in this study. Taken together, our data support that *USP8* pathogenic variants are a marker of worse prognosis in pediatric CD and should be suspected in patients with large invasive corticotropinomas of pediatric onset.

Although we had a limited number of patients with GHE, we elected to include this group of patients in this study to highlight that early onset GHE is mainly driven by known genetics. The mechanism of these tumors involved *GNAS* or *AIP* variants, both genes involved in increased cyclic AMP (cAMP) signaling and activation of the protein kinase A (PKA). The largest study to date on pituitary GHE has studied only germline genetic causes and reported a genetic defect in less than half of the cases [8]. It is possible that some of the remaining cases are explained by somatic variants but that analysis was not presented in the study.

The use of WGS vs. WES for the identification of genetic causes of disorders has been more popular in recent years. We benefited from a collaboration with the NIH Centralized Sequencing Program that offers research genetic testing with WGS for patients enrolled at the NIH and opted to do the same technique at the research somatic sequencing to have comparable results. WGS has the advantage of reviewing untranslated regions which may affect transcriptional regulation of genes that may lead to research findings of novel genetic mechanisms for the pathogenesis of PAs. Additionally, WGS offers reliable CNV analysis which has emerged in recent years as a marker for aggressive PAs [36]. However, we acknowledge that the utility of clinical WGS/WES in patients with PAs is limited at this time.

There are certain limitations to this study. In some cases (*n* = 10), we did not have access to both germline and tumor tissues, so the final results may be underestimating the frequency of known genetic defects. In certain tumors, we only performed somatic screening for the most commonly identified genetic defects. Thus, we may have missed somatic events on other genes described in CD such as *USP48*, *TP53*, *BRAF* etc. These variants may be detected through whole genome sequencing, which was performed for most tumor samples and yielded no hotspot variants in those genes. Also, the cohort of patients with GHE was small compared to patients with CD; larger cohorts of pediatric GHE would be needed to extract genotype-phenotype correlations. However, we have one of the largest cohorts of pediatric patients with CD and present here data on patients that have not been previously reported. Finally, our cohort does not include prolactin-secreting tumors which are the most common type of functional PAs. Our center is mainly a referral center for surgical or rare tumors and since prolactinomas commonly respond to medical therapy, we do not have a large number of these patients/tumors.

In conclusion, genetics of pediatric PAs, especially corticotroph tumors remain unclear. The most commonly identified genetic defect (*USP8* somatic pathogenic variants) is a marker of worse presentation and prognosis. Further research on novel candidate genes is needed for pediatric patients with CD.

## Electronic supplementary material

Below is the link to the electronic supplementary material.

Supplementary Material 1

## Data Availability

Restrictions apply to the availability of some or all data generated or analyzed during this study to preserve patient confidentiality or because they were used for ongoing studies. The corresponding author will on request detail the restrictions and any conditions under which access to some data may be provided.Germline DNA sequencing data are deposited in dpGAP according to NIH regulations and protocol details.
